# Retinal and Choroidal Changes in Children with Moderate-to-High Hyperopia

**DOI:** 10.1155/2021/9971564

**Published:** 2021-09-30

**Authors:** Yu Qian, Yingyan Ma, Qiurong Lin, Zhaoyu Xiang, Jun Qiang, Yan Xu, Haidong Zou

**Affiliations:** ^1^Department of Ophthalmology, Shanghai General Hospital, Shanghai Jiao Tong University School of Medicine, Shanghai, China; ^2^National Clinical Research Center for Eye Diseases, Shanghai Key Laboratory of Ocular Fundus Diseases, Shanghai Engineering Center for Visual Science and Photomedicine, Shanghai Engineering Center for Precise Diagnosis and Treatment of Eye Diseases, Shanghai, China; ^3^Shanghai Eye Diseases Prevention & Treatment Center, Shanghai Eye Hospital, Shanghai, China

## Abstract

**Purpose:**

This study aimed to investigate the characteristics of retinal nerve fiber layer (RNFL) thickness, ganglion cell layer (GCL) thickness, and choroidal thickness in children with moderate-to-high hyperopia (MHH).

**Methods:**

This was a cross-sectional study that enrolled 53 children with MHH and 53 emmetropic children. Subjects with a spherical equivalent refraction (SER) of +4.0 D or higher were included in the MHH group, and subjects with SER between −1.0 D and +1.0 D were included in the emmetropic group. Ophthalmic examinations, including uncorrected visual acuity, cycloplegic refraction, slit-lamp examination, axial length, and swept-source optical coherence tomography (SS-OCT; DRI OCT Triton-1, Topcon, Tokyo, Japan), were performed.

**Results:**

The RNFL and GCL in the temporal and inferior quadrants in 1–3 mm of the macular fovea were thinner in the MHH group than in the emmetropic group (all *P* < 0.05). The MHH group also had a thicker choroidal thickness in all regions (all *P* < 0.05). The SER was independently correlated with the average choroidal thickness in the optic disc and fovea (coefficient = 4.853, *P* < 0.001 for the optic disc; coefficient = 5.523, *P*=0.004 for the fovea), while axial length was negatively correlated with choroidal thickness (coefficient = −12.649, *P* < 0.001). Axial length was positively associated with RNFL and GCL thickness in the temporal quadrant in 1–3 mm of the macular fovea (coefficient = 0.966, *P*=0.007 for RNFL and coefficient = 1.476, *P*=0.011 for the macular fovea).

**Conclusion:**

Compared with emmetropic children, MMH children had greater choroidal thickness. The characteristics of the RNFL and GCL thickness in MMH children were different from those in emmetropic children.

## 1. Introduction

The refractive status of healthy children gradually changes from hyperopia to emmetropia [1]. However, in approximately 3.2% of children [2], the spherical equivalent refraction (SER) is maintained at moderate-to-high hyperopia (MHH) (SER ≥ 4.00 D) [2] and the speed of emmetropization is very slow [3]. Because it is difficult to form a clear image in the retinal plane under hyperopic conditions, such children often have poor vision. Furthermore, as the eyeball is smaller and the iris and lens are thicker and located more anteriorly, the risk of the peripheral iris tissue blocking the anterior chamber angle is higher. This makes MHH an additional risk factor for angle-closure glaucoma (ACG) [4, 5]. Once it develops, ACG can cause irreversible vision loss. However, the development of glaucoma in children is insidious; it usually affects younger children and leads to few complaints. Moreover, visual field examination cannot be performed easily and the measurement of intraocular pressure is affected by corneal curvature, central corneal thickness, and corneal biomechanics [6], making the early diagnosis of glaucoma difficult.

In addition to intraocular pressure and visual field examination, thinning of the retinal nerve fiber layer (RNFL) (including the pRNFL) and the ganglion cell layer (GCL) (including the macular ganglion cell layer) [7] is also known to provide important evidence suggestive of a diagnosis of glaucoma. However, the existing database of RNFL and GCL thickness in children is based on data from children with emmetropia or low and moderate diopter SER [8–10]. In our opinion, this database may not be suitable for the diagnosis of glaucoma in children with MHH. Tas et al. [11] and Dikkaya and Karaman Erdur [12] used Stratus optical coherence tomography (OCT) and spectral domain OCT, respectively, and found that the pRNFL of children with high hyperopia was thicker than that of children with low hyperopia. However, these results remain controversial. For example, Wenner et al. [13] used OCT to measure the pRNFL of children in different refractive states and showed that there was no statistical difference between children with MHH and children with −1.0 to 3.0 D vision. Dikkaya and Karaman Erdur [12] found no difference in inner macular GCL (mGCL) thickness between the two groups. There are only a few reports of RNFL and GCL thickness in MHH children, and all existing data are from European populations. To date, a growing number of researchers have suggested that the choroid in angle-closure glaucoma is thicker, and an increase in choroidal volume may cause a decrease in the anterior segment space [14–16]. However, there is still a lack of data on choroidal thickness in MHH children.

In our study, RNFL, GCL, and choroid thickness in MHH and emmetropic children were measured using SS-OCT, and the differences between the two groups were compared. This study aimed to explore characteristics of the changes in RNFL, GCL, and choroidal thickness in MHH children.

## 2. Patients and Methods

This hospital-based case-control study was approved by the ethics committee of Shanghai General Hospital, Shanghai Jiao Tong University School of Medicine (approval number: No. 2020KY018), and conducted in accordance with the ethical standards stipulated in the Declaration of Helsinki in 1964. After obtaining informed consent from the subjects or legal guardians of these children, the children were examined and their personal data were strictly protected.

From October 2019 to January 2020, the subjects were selected from children who were outpatients at the Shanghai Eye Disease Prevention and Treatment Center. The inclusion criteria were as follows: (1) MHH group: SER ≥ 4.0 D in either eye; (2) emmetropic group: −1.0 D ≤ SER ≤ 1.0 D in either eye; (3) intraocular pressure in the range of 10–21 mmHg; and (4) age < 18 years. The exclusion criteria were as follows: (1) an earlier history of eye disease, such as strabismus, corneal disease, lens disease, glaucoma, maculopathy, and other eye diseases, that may cause retinopathy; (2) a history of ocular surgery; (3) presence of systemic diseases such as hypertension or diabetes; and (4) uncooperative during the examination or the OCT image obtained not being clear and unable to be used for analysis.

An experienced clinical ophthalmologist (Y. Q.) recorded the age, height, weight, and other basic information of the subjects and completed the following ophthalmic examinations: uncorrected visual acuity test, slit-lamp biomicroscopy (SL130, Zeiss, Germany) examination; axial length (AL) was measured using an IOL Master 700 (Carl Zeiss Meditec, Dublin, CA, USA). After pupil dilation, the diopter was measured using an automatic refractometer (KR-8900, Topcon, Tokyo, Japan). An experienced optometrist conducted subjective optometry of the subjects. The best-corrected visual acuity (BCVA) was assessed using the international standard logMAR visual chart.

**Figure 1:**
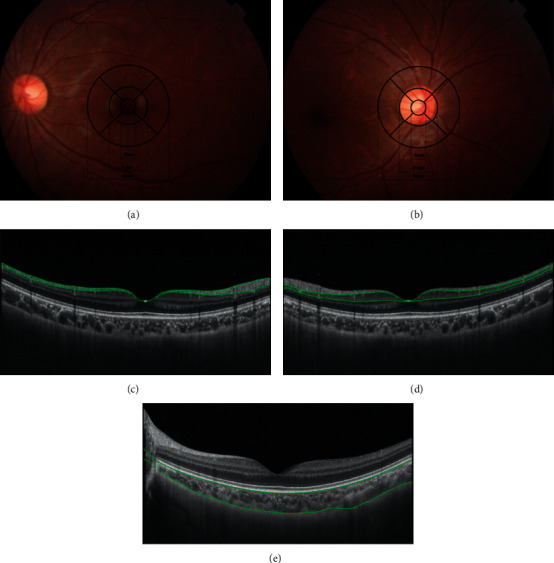
Optical coherence tomography schematic diagram. (a) The Early Treatment Diabetic Retinopathy Study (ETDRS) grid: central circle (diameter = 1 mm), macular inner circle (diameter = 3 mm), and macular outer circle (diameter = 6 mm). (b) ETDRS grid: central circle (diameter = 1 mm), optic disc inner circle (diameter = 3 mm), and optic disc outer circle (diameter = 6 mm). (c) RNFL thickness (the interface between the nerve fiber layer and ganglion cell layer to the inner limiting membrane). (d) GCL thickness (the interface between the nerve fiber layer and ganglion cell layer to the interface between the inner plexiform layer and inner core layer). (e) Choroidal thickness (Bruch's membrane to the choroid-sclera interface).

SS-OCT (DRI OCT Triton-1, Topcon, Tokyo, Japan) was used to evaluate the retina and choroid of the subjects. The optic disc center and the macular fovea were taken as center points with a 6 mm diameter, and 12-line radial scan patterns with a resolution of 1,024 DPI were used to scan the optic disc and macula. The optic disc and macula were divided into three rings with diameters of 1 mm (center), 3 mm (inner), and 6 mm (outer) (Figures 1(a) and 1(b)). The inner and outer rings were divided into four quadrants: temporal, superior, nasal, and inferior. The thickness of each layer of the retina and choroid was analyzed using the built-in software of the SS-OCT device. In this study, RNFL (the interface between the nerve fiber layer and ganglion cell layer to the inner limiting membrane), GCL (the interface between the nerve fiber layer and ganglion cell layer to the interface between the inner plexiform layer and inner core layer), and choroid (Bruch membrane to the choroid-sclera interface) thicknesses were selected for analysis (Figures 1(c)–1(e)).

The sample size was calculated according to the mean and standard deviation of pRNFL thickness in high and low hyperopic children in Dikkaya's and Karaman Erdur study [12], using a two-sample *t*-test, which allowed for unequal variance following the method in PASS 15.0.5 (NCSs, LLC). The parameters were *µ*_1_ (113.2 *µ*m), *σ*_1_ (13.1 *µ*m), *µ*_2_ (101.4 *µ*m), *σ*_2_ (7.0 *µ*), *α* (0.05), and power (0.9). In this study, the ratio of the size of the MHH group to that of the emmetropic group was 1 : 1, and the basic sample size requirement of each group was 18 eyes.

Body mass index (BMI) was calculated as body weight (kg)/height^2^ (m^2^). SER was calculated as spherical power +1/2 cylindrical power. For the MHH and control groups, the data from any eye that met the requirements of this study were included in the statistical analysis.

SPSS (version 23.0; SPSS for Windows, Chicago, IL, USA) was used for all statistical analyses. Continuous variables were presented as mean ± standard deviation, and categorical data were presented as rates (proportions). The data distribution was examined using the Kolmogorov–Smirnov test; Levene's test was used to examine the variance of the data. If the data were normally distributed and had homogeneous variance, the data were analyzed using the two independent samples *t*-test; otherwise, the Mann–Whitney test was used. The chi-square test was used to analyze the categorical data. The independent factors related to RNFL, GCL, and choroidal thickness were determined using stepwise multiple linear regression analysis.

## 3. Results

There were 53 children (19 males, 34 females) with MHH who met the inclusion criteria. Another 53 children (27 male, 26 female) with emmetropia in the same period were also recruited to the emmetropic group. The sex and age of the two groups were matched. Compared with the emmetropic group, the MHH group had significant differences in axial length, SER, BMI, and BCVA (Table 1).

The thickness of the pRNFL was generally greater in the MHH group than in the emmetropic group (Table 2). However, except for the macular fovea, which was significantly thicker in the MHH group than in the emmetropic group, the MHH group had a significantly thinner RNFL than the emmetropic group in the temporal and inferior quadrants in the inner ring of the macula (Table 3). The thickness of the GCL in the optic disc area of the MHH group was generally greater than that of the emmetropic group (Table 2). However, the GCL in the temporal and inferior quadrants of the inner macula in the MHH group were thinner than those in the emmetropic group. The outer macula in the MHH group was thicker and the average thickness of the macula was greater than that in the emmetropic group (Table 3). All quadrants and the average thickness of the choroid in the macula and optic disc in the MHH group were significantly greater than those in the emmetropic group (Tables 2 and 3).

The significantly correlated factors in the single-factor analysis included sex, age, BMI, SER, and AL. These factors were used as dependent variables in the stepwise multiple linear regression analysis. The results showed that there was no correlation between the thickness of the GCL and RNFL in the fovea and the selected independent variables. SER was an independent factor for pRNFL thickness and average choroidal thickness in the optic disc and fovea. Axial length was negatively correlated with mGCL and choroidal thickness in the macula. Age was positively associated with pRNFL and GCL in the optic disc (Table 4).

## 4. Discussion

This study indicated that the RNFL and the GCL in the inferior and temporal quadrants in the 1 mm to 3 mm diameter of the macular fovea in MHH children were thinner than those in emmetropic children, and the choroid in MHH children was generally thicker than that in emmetropic children. To the best of our knowledge, these results have not been reported in previous studies.

Regarding the characteristics of RNFL thickness, our research found that the pRNFL in the temporal quadrant was significantly thicker in the MHH group than in the emmetropic group. This is similar to the results reported by Tas et al. [11], Dikkaya and Karaman Erdur [12], and Wenner et al. [13]. Their studies suggested that, compared to an emmetropic group, the pRNFL in MHH children is significantly thicker, including the average pRNFL and inferior pRNFL. In terms of mGCL thickness, Dikkaya and Karaman Erdur [12] reported that MHH children had a thinner GCL in the nasal, temporal, and inferior areas of the outer macula. Our results are different in that the mGCL was thinner in the temporal and inferior quadrants of the inner macula and thicker in the outer macula in MHH children than in emmetropic children. This difference may be due to differences in the ethnicities of the study populations.

To date, it is not clear whether the thickness of the RNFL and GCL in MHH children is increased or decreased. This may be due to the influence of retinal development in children with MHH. The macula, which is located in the temporal part of the optic disc, contains a large number of RGCs (retinal ganglion cells) and nerve fiber bundles. It has been confirmed that RGC cells in the macula gradually migrate from the fovea to the surrounding area with the development of the fovea; RGC development in the temporal area of the macula is relatively immature at birth, and the process of migration stops when the fovea matures [17]. A possible cause for the observed discrepancies in MHH children might be that a lack of clear visual stimulation influences the development of the macula, which might then result in relatively few RGC migrations. Therefore, compared with that in emmetropic children, the thickness of the GCL and RNFL in the temporal and inferior quadrants may be lower. The thickness of the pRNFL in the temporal and inferior quadrants and the GCL in the outer macula in MHH children might be because the AL in MHH children is shorter; consequently, the eyeball and retinal area are smaller. At the same time, the papillomacular fibers are located in this area and contain a large number of nerve fiber bundles [18]. In contrast, MHH has an effect on the physiological apoptosis of RGCs during the growth and development of children, which might lead to the thickening of the GCL and RNFL in these areas [17].

Due to the characteristic anatomy of the eyeballs of MHH children, the development of glaucoma is possible. In addition to evaluating the thickness of the pRNFL in glaucoma, the thickness of the mGCL can also reflect the damage to macular function in glaucoma [7]. Hood et al. [19] found that, in patients with early glaucoma, the change in the mGCL preceded that in the pRNFL. Hood et al. [20] and Schiefer et al. [21] also found that the mGCL in the inferior quadrant of glaucoma patients was thinner. Children with MHH show a thinner RNFL and GCL in the inner macula in childhood, and this anatomical structure may indicate that they are more likely to suffer from glaucoma in adulthood, which also explains why MHH is a risk factor for glaucoma [22].

The choroid is an important tissue in the eyeball and is rich in blood vessels. Some studies have found that a thicker choroid might affect the process of emmetropization in children with MHH [23]. Troilo et al. showed that choroidal thickening in primates is related to hyperopia [23]. They suggested that normal choroidal thickening is a method of controlling the growth of the eyeball and can appropriately slow the increase in AL during the rapid growth of the eyeball. Our study showed that the choroidal thickness in MHH children was greater, and the average choroidal thickness in the optic disc and macular fovea was higher with a higher SER, while the average thickness of the choroid in the macula decreased with increasing AL. Choroidal thickness was negatively correlated with AL, which is similar to the results of previous studies. In a cross-sectional study of the retina of 276 Chinese children aged 7–13 years, Jin et al. [24] found that choroidal thickness was related to axial length in Chinese children. They believed that, in the early stages of myopia, choroidal thinning occurred before retinal thinning. Kaderli et al. [25] found that the macular choroid thickened with an increase in hyperopia and a decrease in AL. At the same time, their study also found that the diameter and area of blood vessels in the macular choroid also increased. Oner et al. [26] found that the choroidal thickness in the fovea decreased with an increase in the AL, and hyperopia was related to the choroidal thickness in the fovea, while amblyopia had no independent significant effect on the choroidal thickness in the fovea. Therefore, our results add evidence to the hypothesis that choroidal thickening may play an important role in the stagnation of the emmetropization process in MHH children.

Through stepwise multiple linear regression analysis, SER, AL, and age were found to be significantly positively correlated with RNFL thickness. This may be because the nerve fiber bundles of the upper and lower arch between the optic disc and macula gradually approached the level of the temporal side, resulting in an increase in thickness [27]. There was a positive correlation between the thickness of the mGCL in the temporal and inferior quadrants, AL, and age. It is possible that the number of amacrine cells in the GCL increased with an increase in AL and age [28]. The mGCL thickness in the outer macula was negatively correlated with AL and age, which may be due to the high density of RGCs in the macula. With the increase in AL, the area of the macula increased, and the density of RGCs decreased accordingly. A greater change was found further away from the fovea [29].

One limitation of this study is its cross-sectional design. In future studies, these children will be followed up to observe changes in the RNFL and GCL in children with MHH, and the examination of intraocular pressure, visual field, and anterior segment OCT will be added. Another limitation is that the number of participants was relatively small because of limited access to MMH children. Future studies must include more children with MHH.

## 5. Conclusions

Compared with emmetropic children, children with MHH had greater choroidal thickness. The characteristics of RNFL and GCL thickness in MMH children were different from those in emmetropic children.

## Figures and Tables

**Table 1:** Descriptive and ocular parameters of the MHH and emmetropic groups.

	MHH group *n* = 53	Emmetropic group *n* = 53	*P* value
Age (y)	6.83 ± 2.16	7.45 ± 1.82	0.067^∗^
*N* (male sex)	53 (19)	53 (27)	0.117^†^
SER (D)	6.38 ± 1.64	−0.17 ± 0.68	<0.001^∗^
Axial length (mm)	20.90 ± 0.99	23.59 ± 0.96	<0.001^‡^
BMI	16.23 ± 2.85 (*n* = 44)	16.74 ± 2.72 (*n* = 51)	<0.001^∗^
BCVA (logMAR)	0.19 ± 0.19 (*n* = 52)	0.02 ± 0.07	<0.001^∗^

D: diopter; results are mean ± SD or *n* (%); ^∗^Mann–Whitney *U* test; ^†^chi-square test; ^‡^independent samples *t*-test.

**Table 2:** Comparison of the thickness of the RNFL, GCL, and choroid in the disc between the MHH and emmetropic groups.

	Layer	MHH group	Emmetropic group	*P* value
TI	RNFL	111.67 ± 23.24	100.66 ± 20.40	0.010^∗^
	GCL	46.18 ± 11.96	45.02 ± 11.31	0.228^∗^
	Choroid	127.18 ± 41.49	107.75 ± 51.82	0.006^∗^

SI	RNFL	181.58 ± 30.77	171.94 ± 32.00	0.136^∗^
	GCL	38.74 ± 11.85	33.85 ± 13.03	0.048^†^
	Choroid	105.12 ± 41.13	90.25 ± 44.24	0.029^∗^

NI	RNFL	140.96 ± 33.71	130.96 ± 35.85	0.146^†^
	GCL	38.66 ± 11.31	36.77 ± 12.66	0.140^∗^
	Choroid	93.87 ± 43.25	83.13 ± 54.07	0.043^∗^

II	RNFL	198.09 ± 33.22	178.53 ± 26.23	0.001^†^
	GCL	33.50 ± 13.05	32.74 ± 8.04	0.617^∗^
	Choroid	112.46 ± 49.93	92.20 ± 44.95	0.005^∗^

TO	RNFL	66.14 ± 12.68	62.88 ± 12.82	0.050^∗^
	GCL	59.92 ± 9.69	63.42 ± 8.59	0.053^†^
	Choroid	202.48 ± 42.97	150.38 ± 44.76	<0.001^†^

SO	RNFL	116.73 ± 16.08	112.22 ± 13.74	0.127^†^
	GCL	37.70 ± 5.18	37.95 ± 6.10	0.818^†^
	Choroid	169.79 ± 43.17	132.24 ± 35.28	<0.001^†^

NO	RNFL	65.81 ± 21.21	61.12 ± 19.35	0.162^∗^
	GCL	40.99 ± 5.79	41.03 ± 6.90	0.268^∗^
	Choroid	163.14 ± 54.98	132.45 ± 46.47	0.003^†^

IO	RNFL	115.40 ± 16.91	100.12 ± 14.85	<0.001^∗^
	GCL	37.86 ± 5.97	35.60 ± 7.23	0.055^∗^
	Choroid	159.05 ± 48.33	119.51 ± 37.57	0.014^∗^

Average	RNFL	101.47 ± 8.71	94.53 ± 7.55	<0.001^∗^
	GCL	43.36 ± 4.12	43.29 ± 5.18	0.939^†^
	Choroid	163.13 ± 36.76	126.96 ± 37.01	<0.001^†^

Results are mean ± SD; ^∗^Mann–Whitney *U* test; ^†^independent samples *t*-test. II: inferior sector of the inner ring; IO: inferior sector of the outer ring; NI: nasal sector of the inner ring; NO: nasal sector of the outer ring; SI: superior sector of the inner ring; SO: superior sector of the outer ring; TI: temporal sector of the inner ring; TO: temporal sector of the outer ring.

**Table 3:** Comparison of the thickness of the RNFL, GCL, and choroid in the macula between the MHH and emmetropic groups.

	Layer	MHH group	Emmetropic group	*P* value
Subfoveal	RNFL	9.48 ± 5.66	7.25 ± 4.84	0.028^a^
	GCL	52.49 ± 14.66	49.80 ± 12.22	0.172^a^
	Choroid	292.33 ± 66.92	241.42 ± 55.51	<0.001^c^

TI	RNFL	19.85 ± 4.64	22.26 ± 6.34	0.003^a^
	GCL	80.48 ± 10.08	85.01 ± 7.75	0.021^a^
	Choroid	297.99 ± 63.58	255.43 ± 57.46	0.001^c^

SI	RNFL	28.97 ± 7.66	29.02 ± 4.98	0.630^a^
	GCL	91.29 ± 8.25	90.68 ± 7.32	0.695^c^
	Choroid	285.50 ± 55.11	245.56 ± 52.19	<0.001^c^

NI	RNFL	26.43 ± 7.59	26.84 ± 8.49	0.428^a^
	GCL	91.76 ± 8.94	91.48 ± 9.10	0.677^a^
	Choroid	284.63 ± 63.89	211.15 ± 50.83	<0.001^c^

II	RNFL	25.89 ± 8.22	28.76 ± 9.75	0.014^a^
	GCL	86.41 ± 10.01	88.59 ± 10.40	0.038^a^
	Choroid	290.71 ± 61.92	246.03 ± 57.87	<0.001^c^

TO	RNFL	23.41 ± 3.51	24.39 ± 5.79	0.445^a^
	GCL	76.30 ± 6.59	68.83 ± 8.08	<0.001^c^
	Choroid	283.80 ± 50.86	255.25 ± 52.28	<0.001^c^

SO	RNFL	42.67 ± 6.00	42.20 ± 4.69	0.866^a^
	GCL	69.03 ± 7.28	63.82 ± 6.57	<0.001^c^
	Choroid	278.66 ± 50.09	233.37 ± 44.09	<0.001^c^

NO	RNFL	48.96 ± 9.43	52.62 ± 11.13	0.063^a^
	GCL	74.00 ± 7.74	69.46 ± 8.43	0.001^a^
	Choroid	251.55 ± 50.93	164.19 ± 40.87	<0.001^c^

IO	RNFL	41.96 ± 6.24	43.49 ± 8.51	0.348^a^
	GCL	70.86 ± 8.92	62.74 ± 7.09	<0.001^c^
	Choroid	271.19 ± 53.47	228.74 ± 47.30	<0.001^c^

Average	RNFL	35.32 ± 3.92	36.61 ± 4.33	0.154^a^
	GCL	75.31 ± 5.12	70.86 ± 4.78	<0.001^c^
	Choroid	275.99 ± 45.13	225.31 ± 42.83	<0.001^c^

Results are mean ± SD; ^a^Mann–Whitney *U* test; ^c^independent samples *t*-test. II: inferior sector of the inner ring; IO: inferior sector of the outer ring; NI: nasal sector of the inner ring; NO: nasal sector of the outer ring; SI: superior sector of the inner ring; SO: superior sector of the outer ring; TI: temporal sector of the inner ring; TO: temporal sector of the outer ring.

**Table 4:** Correlations between the retina, RNFL, ganglion cell layer, and choroid thickness and spherical equivalent, age, and axial length.

		Independent factor	Unstandardized coefficients		SC	*P* value	*r* ^2^
			*B*	SD	*β*		
pRNFL	TI	SER	4.966	1.247	0.807	<0.001	0.159
		AL	7.911	2.644	0.607	0.004	
	II	SER	4.326	0.756	0.507	<0.001	0.319
		Age	5.676	1.400	0.359	<0.001	
	IO	SER	2.292	0.454	0.468	<0.001	0.219
	Average	SER	1.222	0.245	0.467	<0.001	0.238
		Age	1.214	0.454	0.250	0.009	

mRNFL	TI	AL	0.966	0.351	0.277	0.007	0.077

pGCL	SI	Age	−1.970	0.669	−0.295	0.004	0.087

mGCL	TI	AL	1.476	0.568	0.263	0.011	0.069
	SI	Age	1.106	0.552	0.206	0.048	0.042
	TO	AL	−2.436	0.454	−0.490	<0.001	0.240
	SI	AL	−2.035	0.418	−0.455	<0.001	0.207
	NO	AL	−1.679	0.499	−0.333	0.001	0.111
	IO	AL	−2.746	0.473	−0.520	<0.001	0.270
	Average	Age	0.599	0.270	0.211	0.029	0.268
		AL	−1.783	0.311	−0.545	<0.001	

pCH	Average	SER	4.853	1.075	0.428	<0.001	0.183

mCH	Fovea	SER	5.523	1.852	0.298	0.004	0.089
	Average	AL	−12.649	2.874	−0.419	<0.001	0.175

II: inferior sector of the inner ring; IO: inferior sector of the outer ring; NI: nasal sector of the inner ring; NO: nasal sector of the outer ring; SI: superior sector of the inner ring; SO: superior sector of the outer ring; TI: temporal sector of the inner ring; TO: temporal sector of the outer ring; pCH: peripapillary choroid; mCH: macular choroid; pRNFL: peripapillary retinal nerve fiber layer; mRNFL: macular retinal nerve fiber layer; pGCL: peripapillary ganglion cell layer; mGCL: macular ganglion cell layer.

## Data Availability

In our study, the children were examined, and their personal data were strictly protected, after obtaining informed consent of the subjects or legal guardians of these children. Therefore, all the original data are stored in Shanghai Eye Disease Prevention and Treatment Center. If necessary, we can e-mail all the original data to the editorial office.

## Additional Points

In brief, we found that RNFL and GCL thicknesses in the temporal and inferior quadrants in the 1 mm to 3 mm diameter of the macular fovea were lesser, and all parts of the choroidal thickness were greater in children with moderate-to-high hyperopia than in emmetropic children.
